# Prognostic and clinicopathological value of circPVT1 in human cancers: A meta‐analysis

**DOI:** 10.1002/cnr2.1385

**Published:** 2021-04-01

**Authors:** Zhengjun Lin, Xianzhe Tang, Lu Wang, Lin Ling

**Affiliations:** ^1^ Department of Orthopedics The Second Xiangya Hospital Central South University Changsha China; ^2^ Xiangya School of Medicine Central South University Changsha China; ^3^ Department of Orthopedics Chenzhou No.1 people's Hospital Chenzhou China

**Keywords:** cancer, circular RNA PVT1, meta‐analysis, prognosis

## Abstract

**Background:**

Circular RNA PVT1 (circPVT1) is significantly upregulated in various human cancers and is related to poor clinical outcome of cancer patients. However, the prognostic and clinicopathological value of circPVT1 in diverse human cancers remains controversial and inconclusive.

**Aim:**

The objective of our study is to evaluate the prognostic and clinicopathological role of circPVT1 for cancer patients.

**Methods and results:**

PubMed, Embase, Web of Science, and Cochrane Library were searched for eligible studies by October 1, 2020. The correlation between circPVT1 expression, and overall survival (OS) and clinical parameters was assessed by pooled hazard ratios (HRs) and odds ratios (ORs) with 95% confidence intervals (CIs). Subgroup analyses, heterogeneity, and publication bias were conducted to further enhance reliability. Twelve studies (1282 patients) were selected for this meta‐analysis, including 11 on prognosis and 10 on clinicopathological parameters. Elevated expression of circPVT1 was associated with a worse OS in cancer patients (HR, 2.009; 95% CI, 1.667‐2.408, 1.892; *P* < .001). For clinicopathological value, upregulation of circPVT1 was closely related to poor clinical parameters lymph node metastasis (OR = 2.019; 95% CI, 1.026‐3.976; *P* = .042; *I* ^2^ = 77.5%; P_H_ = 0.000), late clinical stage (OR = 3.594; 95% CI, 1.828‐7.065; *P* < .001; *I* ^2^ = 71.7%; P_H_ = 0.001), distant metastasis (OR = 4.598; 95% CI, 1.411‐14.988; *P* = .011; *I* ^2^ = 78.1%; P_H_ = 0.001), and chemoresistant (OR = 6.400; 95% CI, 2.107‐19.441; *P* = .001; *I* ^2^ = 49.6%; P_H_ = 0.159).

**Conclusion:**

High expression of circPVT1 is correlated with unfavorable prognosis of cancer patients, indicating that circPVT1 can function as a potential prognostic biomarker in human cancer.

## 1 BACKGROUND

Circular RNAs (circRNAs) comprise a novel class of non‐coding RNAs with a closed‐loop structure and tissue‐specific expression pattern in eukaryotes.^1^ In contrast to the classical canonical splicing of mRNAs, circRNAs are produced by a unique back‐splicing process, and circRNA biogenesis is regulated by various cis‐elements and trans‐factors.^2^ It has been extensively studied that circRNAs can exert important biological functions, including sponging miRNAs, regulating gene expression, interacting with proteins, and even producing proteins.^3^ With the development of experimental technologies, such as high‐throughput RNA sequencing, an increasing number of circRNAs has been detected in the past decades.^4^, ^5^, ^6^ Notably, numerous works have indicated that circRNAs play pivotal roles in various physiological processes and diverse human diseases, including human cancer.^7^, ^8^, ^9^ It has been found that a great number of circRNAs are dysregulated in various human cancers and play an important role in tumor progression.^10^ Furthermore, the promising roles of several circRNAs as prognostic and diagnostic biomarkers in human cancer have been reported in recent studies. For example, it has been found that circHIPK3 can serve as a diagnostic biomarker for osteosarcoma, and downregulation of circHIPK3 is associated with poor OS of osteosarcoma patients.^11^

Circular RNA PVT1 (circPVT1), also named hsa_circ_0001821, is located on chromosome 8q24 and is derived from the *PVT1* gene locus.^12^ Very recent works have proved that circPVT1 is evidently upregulated in multiple cancer types and serves as an oncogenic non‐coding RNA during cancer development. CircPVT1 has been found to promote proliferation, metastasis, and chemoresistance in several human cancers.^13^ For example, circPVT1 can facilitate cell proliferation and inhibit apoptosis via sponging miR‐497 in non‐small cell lung cancer (NSCLC) in both vivo and vitro.^14^ It has also been found that circPVT1 has a great diagnostic accuracy for some cancer types, such as NSCLC, gastric cancer (GC), and oral squamous cell carcinoma (OSCC).^15^, ^16^, ^17^, ^18^, ^19^ Moreover, emerging evidence has shown that the upregulation of circPVT1 is correlated with poor OS in several types of human cancer, such as lung adenocarcinoma (LAD), breast cancer (BCa), and GC.^15^, ^20^, ^21^ Besides, the clinicopathological value of circPVT1 in human cancers has also been identified in multiple studies.^18^ However, the certain prognostic and clinicopathological value of circPVT1 in human cancers is still inclusive and some studies provided contradictory outcomes. For instance, Chen et al^15^ and Wang et al^22^ demonstrated that upregulation of circPVT1 was significantly associated with lymph node metastasis in GC and colorectal cancer (CRC), respectively; on the contrary, Kong et al^18^ detected that the correlation between circPVT1 expression and lymph node metastasis in GC was negative. Similarly, it was found that elevated expression of circPVT1 was related to tumor size in NSCLC and hepatocellular carcinoma (HCC); in contradiction to these studies, studies of Yan et al^23^ and Bian et al^20^ indicated that there was no evident relationship between circPVT1 expression and tumor size. Therefore, we conducted the following meta‐analysis to assess the prognostic and clinicopathological value of circPVT1 in human cancers, thus highlighting the potential role of circPVT1 as clinical biomarker and novel therapy target for cancer.

## 2 METHODS

### 2.1 Data search strategy

The present study was performed according to the Preferred Reporting Items for Systematic Reviews and Meta‐Analysis (PRISMA).^24^ Relevant studies published in English before October 1, 2020 were searched in PubMed, Embase, Web of Science, and Cochrane Library online databases. The key words were as follows: (a) “circular RNA PVT1”or “circPVT1” or “hsa_circ_0001821”; (b) “cancer” or “tumor” or “neoplasm” or “carcinoma” or “sarcoma”. Two researchers (ZJL and XZT) conducted the literature search independently, and reference lists were also searched for possible relevant studies. Each retrieved study was comprehensively checked and identified by the two researchers. Any discrepancies were resolved by the corresponding author (LL).

### 2.2 Inclusion and exclusion criteria

Studies in accordance with the following criteria were included: (a) patients with diagnosis of human cancer; (b) studies examining the correlation between prognosis or clinicopathological features of cancer patients; (c) studies published in English; (d) cohort studies that patients divided into “high circPVT1” and “low circPVT1” group.

On the contrary, these studies were excluded: (a) duplication; (b) studies irrelevant to circPVT1 or human cancer; (c) reviews, case reports, letters, and editorials; (d) studies without available data, or studies that we could not contact the authors/investigators for further clarification or data.

### 2.3 Data extraction and quality assessment

Two researchers (ZJL and LW) extracted the following data from eligible studies: first author name; publication year; country; type of cancer; sample size; detection method; cut‐off value; prognostic parameters of circPVT1 including HRs, corresponding 95% CIs and *P* value; clinicopathological characteristics with age, gender, histological grade, tumor size, T stage, lymph node metastasis, clinical stage, distal metastasis, and chemoresistant. If HRs and 95% CIs were not directly provided, they were extracted from Kaplan‐Meier curves according to Tierney's method.^25^ Excel was used to record all the available information. The guideline of Newcastle‐Ottawa Scale (NOS) was adopted to evaluate the quality of eligible studies. Three perspectives including selection, comparability, and exposure were considered. The NOS scores were ranged from 0 (lowest score) to 9 (highest score). A study with a score ≥ 7 was defined as a study with high quality.^26^ The quality of each eligible study was evaluated by two independent researchers. Any discrepancies were resolved by the corresponding author (LL).

### 2.4 Statistical analysis

Statistical analysis was performed using STATA 12.0 software. The correlation between circPVT1 expression and clinicopathological characteristics was evaluated by ORs and 95% CIs. The HR and 95% CI were directly extracted from the original publications. If the study only provided Kaplan‐Meier survival curve, HR and 95% CI were synthesized by Engauge Digitizer 11.2 software (http://markummitchell.github.io/engauge-digitizer/) according to Tierney's method.^25^ Higgin's *I* ^2^ statistics and Cochran's *Q* test were used to identify the heterogeneity of the included studies. If *I* ^2^ ≤ 50% and *P* > .05, a fixed‐effect model test was utilized to analyze the results. Otherwise, a random‐effect model test was applied for analysis.^27^ HR > 1 indicates the poor prognosis for high circPVT1expression level, and *P* < .05 was considered to be statistically significant. Publication bias was qualitatively determined using funnel plot analysis, and quantified by Egger's test and Begg's test. Subgroup analysis and sensitivity analysis were conducted to evaluate the source of heterogeneity and stability of the results.

## 3 RESULTS

### 3.1 Search results

**FIGURE 1:**
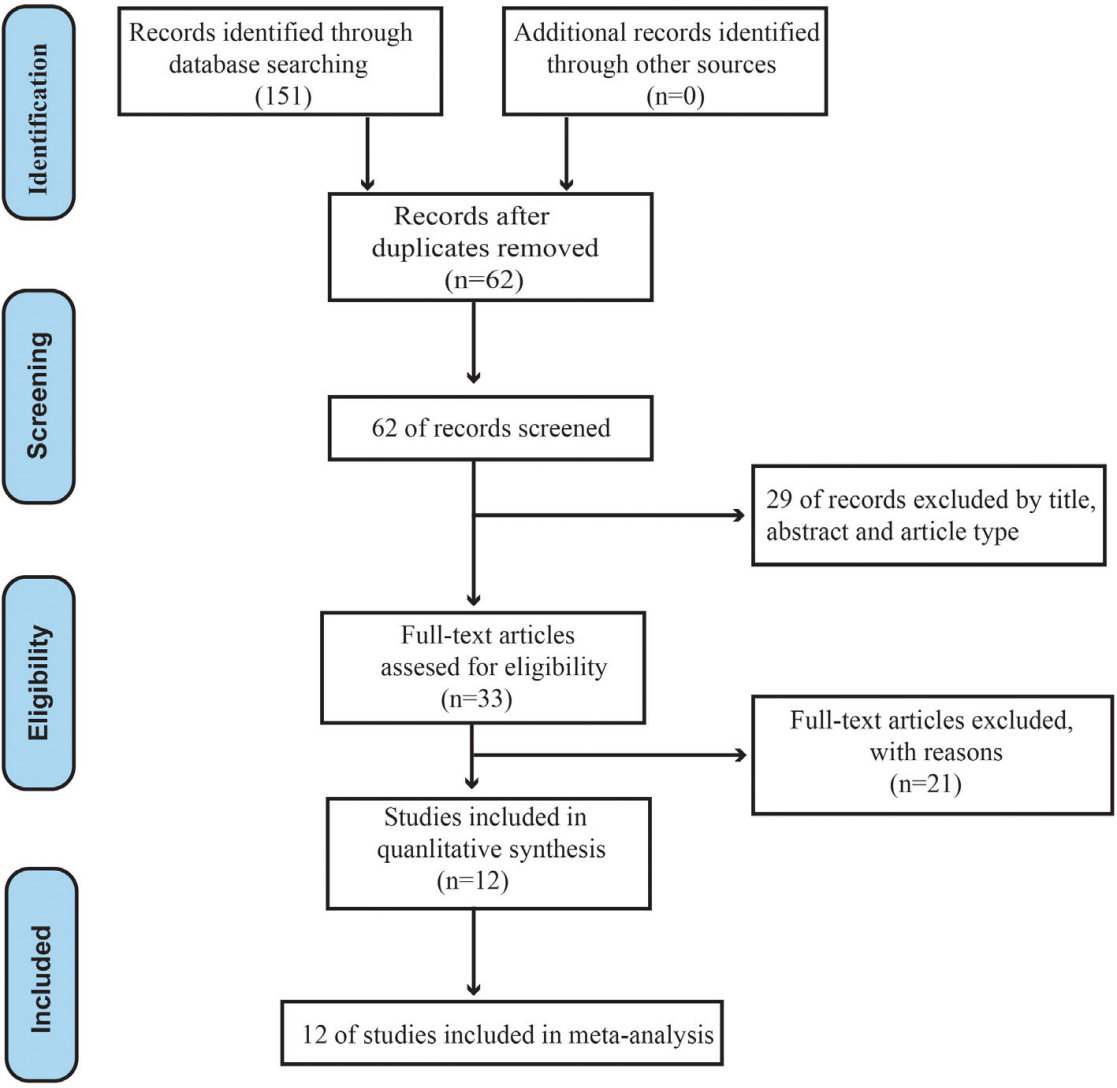
Flow diagram of the literature selection process

One hundred and fifty‐one relevant studies were retrieved after searching database key words. Eighty‐nine studies were excluded based on duplication criteria. After title, abstract, and article type screening, 29 studies were further excluded. Then, based on criteria, 21 articles were excluded for: not focused on circPVT1 or cancer, not reported relevant results, not studied research studies, and no sufficient data. In summary, 12 eligible studies were included in the meta‐analysis (Figure 1).

### 3.2 Study characteristics

A total of 1282 patients were comprised in studies published between 2017 and 2020. The sample size ranged from 48 to 379. The studies involved various cancer types, including lung adenocarcinoma (LAD), non‐small cell lung cancer (NSCLC), breast cancer (BCa), head and neck squamous cell carcinoma (HNSCC), hepatocellular carcinoma (HCC), colorectal cancer (CRC), osteosarcoma, papillary thyroid carcinoma (PTC), and gastric cancer (GC). The circPVT1 expression was measured by qRT‐PCR in all studies. Eleven studies were performed to analyze OS. Three included studies directly provided HR and 95% CI data while remaining eight studies only provided Kaplan‐Meier survival curves. Meanwhile, 10 studies were performed to analyze the clinicopathological parameters. The NOS score of 11 studies was ≥7, indicative of high quality of the majority of the studies included in our research (Table 1).

**TABLE 1:** Characteristics of the studies included in the meta‐analysis

Study	Year	Cancer	Sample	CircPVT1 expression		Survival	HR (95% CI)	*P* value	HR obtained	Measure	Cutoff value	NOS score
				High	Low							
Lorena et al^28^	2017	HNSCC	379	280	89	OS	1.81 (1.11‐2.94)	.017	Direct	qRT‐PCR	Median value	8
Chen et al^15^	2017	GC	183	107	80	OS	1.73 (1.24‐2.42)	.0005	Curve	qRT‐PCR	Youden's index	8
Zhu et al^16^	2018	Osteosarcoma	80	30	50	OS	2.54 (1.28‐5.02)	.002	Curve	qRT‐PCR	Mean	8
Wang et al^22^	2019	CRC	64	32	32	OS	3.20 (1.51‐6.76)	.0005	Curve	qRT‐PCR	Median value	7
Zhu et al^29^	2019	HCC	70	35	35	OS	2.04 (0.94‐4.46)	.0235	Curve	qRT‐PCR	Median value	8
Tao et al^30^	2019	PTC	39	21	18	OS	2.23 (0.60‐8.34)	.0392	Curve	qRT‐PCR	Mean	7
Qin et al^14^	2019	NSCLC	90	43	47	OS	1.83 (0.83‐4.03)	<.05	Curve	qRT‐PCR	Median value	8
Bian et al^20^	2019	BCa	99	52	47	OS	1.88 (0.83‐4.26)	.022	Curve	qRT‐PCR	Median value	8
Kong et al^18^	2019	GC	80	18	62	NA	NA	NA	NA	qRT‐PCR	Youden's index	6
Lu et al^31^	2020	NSCLC	96	48	48	OS	4.284 (2.155‐9.624)	.013	Direct	qRT‐PCR	Mean	8
Zheng et al^21^	2020	LAD	104	48	56	OS	1.679 (1.065‐2.638)	.025	Direct	qRT‐PCR	Median value	8
Yan et al^23^	2020	Osteosarcoma	48	24	24	OS	2.21 (0.96‐5.09)	.0053	Curve	qRT‐PCR	NA	7

Abbreviations: 95% CI, 95% confidence interval; BCa, breast cancer; CRC, colorectal cancer; GC, gastric cancer; HCC, hepatocellular carcinoma; HCC, hepatocellular carcinoma; HNSCC, head and neck squamous cell carcinoma; HR, hazard ratio; LAD, lung adenocarcinoma (LAD); NA, not available; NOS, Newcastle‐Ottawa Scale; NSCLC, non‐small cell lung cancer; OS, overall survival; PTC, papillary thyroid carcinoma; qRT‐PCR, quantitative reverse transcription polymerase chain reaction.

### 3.3 Overall survival

**FIGURE 2:**
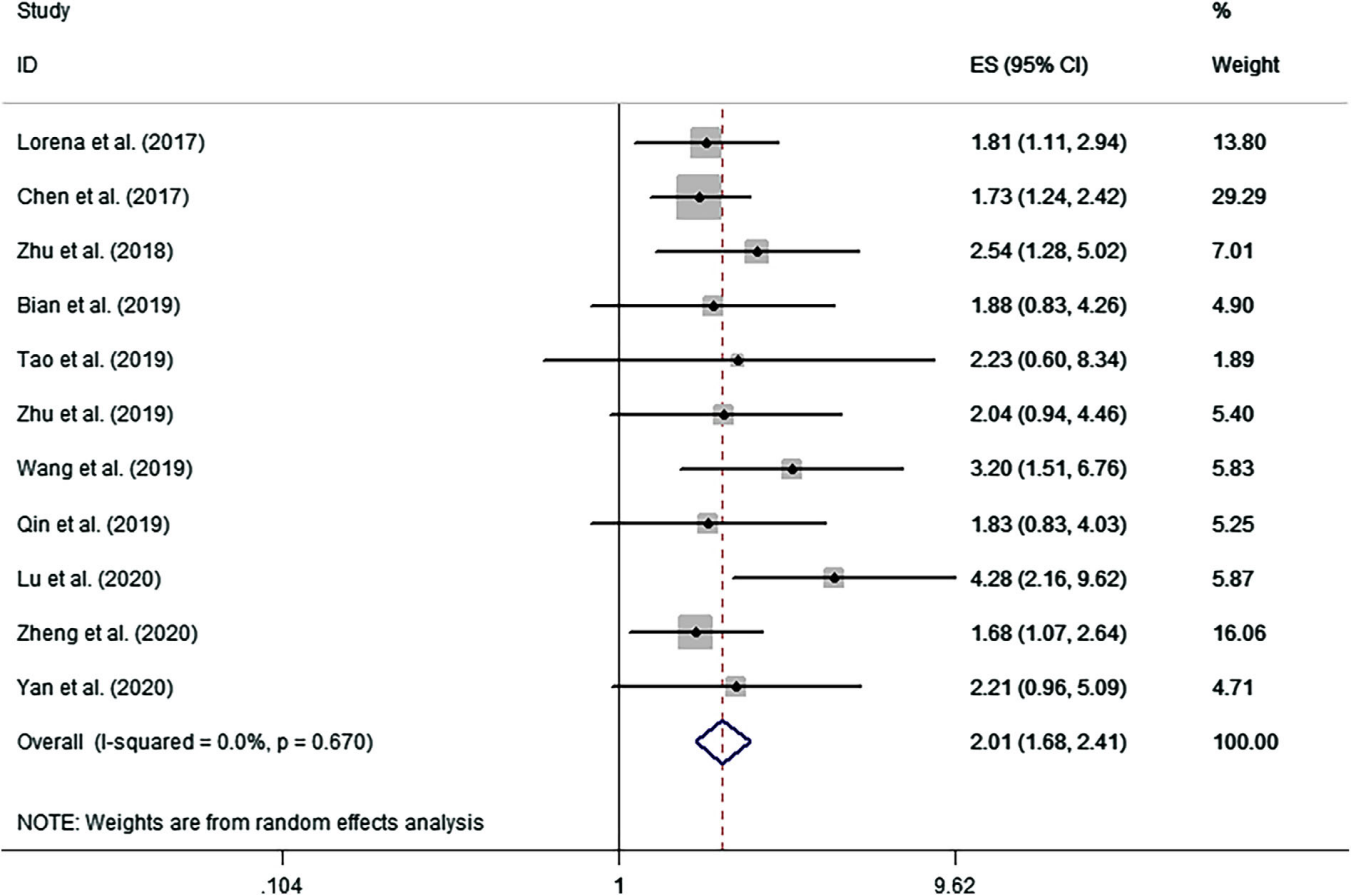
Forest plots assessing the association between overall survival and circPVT1 expression

The combined analysis of published data from 11 analyses indicated that upregulation of circPVT1 was an indicator for poor OS in patients with human cancer (pooled HR, 2.009; 95% CI, 1.667‐2.408; *P* < .001). A fixed‐effects model was selected since no heterogeneity was reported (*I* ^2^ = 0.0%, P_H_ = 0.670, Figure 2).

### 3.4 Subgroup meta‐analyses

**FIGURE 3:**
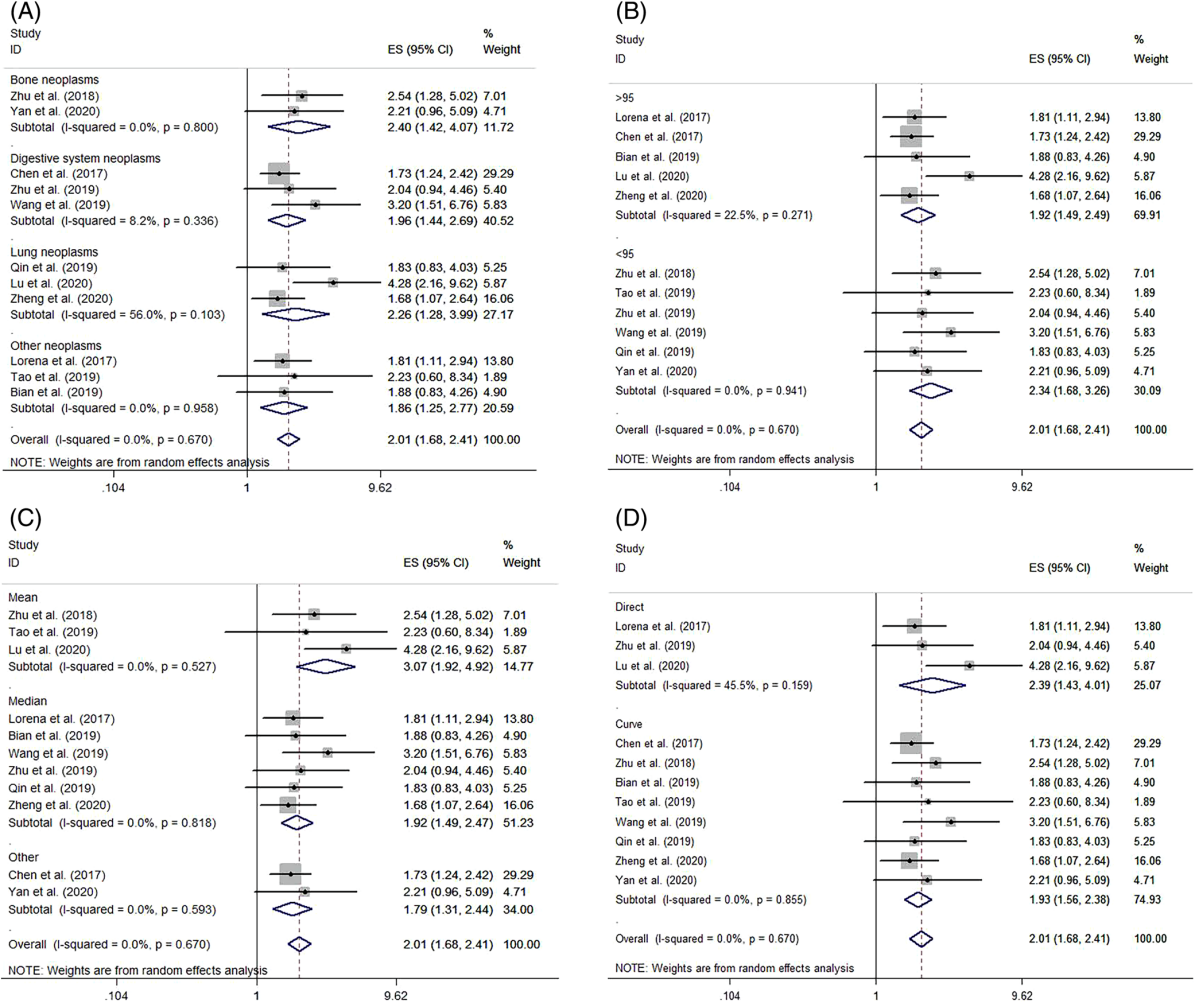
Forest plots of subgroup analyses assessing the association between circPVT1 expression and overall survival based on tumor type (A), sample size (B), different cutoff value (C), and different extraction method (D)

Subgroup meta‐analyses stratified by tumor type (lung neoplasms, bone neoplasms, digestive system neoplasms, and other neoplasms) (Figure 3A), sample size (n ≥ 95 or n < 95) (Figure 3B), cutoff value (median, mean, or others) (Figure 3C), and data‐obtained method (direct or indirect) (Figure 3D) were performed.

#### 3.4.1 Subgroup meta‐analyses based on tumor type

Because there were 9 different tumor types included in 11 studies, we divided them into four subgroups: lung neoplasms, bone neoplasms, digestive system neoplasms, and other neoplasms. The results showed that increased circPVT1 expression could predict unfavorable OS in lung neoplasms (HR = 2.261; 95% CI, 1.283‐3.987; *P* = .005; *I* ^2^ = 56%; P_H_ = 0.103), bone neoplasms (HR = 2.402; 95% CI, 1.426‐4.075; *P* = .001; *I* ^2^ = 0.00%; P_H_ = 0.800), digestive system neoplasms (HR = 1.965; 95% CI, 1.436‐2.689; *P* < .001; *I* ^2^ = 8.2%; P_H_ = 0.336), and other neoplasms (HR = 1.862; 95% CI, 1.249‐2.774; *P* = .002; *I* ^2^ = 0.00%; P_H_ = 0.958) (Figure 3A).

#### 3.4.2 Subgroup meta‐analyses based on sample size

In subgroups divided by sample size, the results showed that in both large sample size (n ≥ 95) (HR = 1.922; 95% CI, 1.485‐2.487; *P* < .001; *I* ^2^ = 22.5%, P_H_ = 0.271) and small sample size (n < 95) (HR = 2.341; 95% CI, 1.683‐3.255; *P* < .001; *I* ^2^ = 0.00%, P_H_ = 0.941), upregulation of circPVT1 was significantly related to worse OS of cancer patients (Figure 3B).

#### 3.4.3 Subgroup meta‐analyses based on cutoff value

Regarding subgroups by different cutoff values, whether median value was used as cutoff value (HR = 1.920; 95% CI, 1.491‐2.472; *P* < .001; *I* ^2^ = 0.00%; P_H_ = 0.818), or mean value was used (HR = 3.074; 95% CI, 1.919‐4.921; *P* < .001; *I* ^2^ = 0.00%; P_H_ = 0.527) or other cutoff values were used (HR = 1.790; 95% CI, 1.312‐2.441; *P* < .001; *I* ^2^ = 0.00%; P_H_ = 0.593), there was close relationship between high circPVT1 expression and poor OS in patients of multiple cancers (Figure 3C).

#### 3.4.4 Subgroup meta‐analyses based on data‐obtained method

Concerning the data‐obtained method subgroups analysis, high circPVT1 expression was correlated with poor OS of cancer patients, whether the data were extracted directly from the studies in the literature (HR = 2.395; 95% CI, 1.430‐4.009; *P* = .001; *I* ^2^ = 45.50%; P_H_ = 0.159) or indirectly from Kaplan‐Meier survival curves in the literature (HR = 1.929; 95% CI, 1.565‐2.377; *P* < .001; *I* ^2^ = 0.00%; P_H_ = 0.855) (Figure 3D).

### 3.5 Clinicopathological parameters

The correlation between circPVT1 and the clinicopathological parameters is shown in Table 2. The outcomes presented the significant correlation between highcircPVT1 expression and lymph node metastasis (OR = 2.019; 95% CI, 1.026‐3.976; *P* = .042; *I* ^2^ = 77.5%; P_H_ = 0.000), late clinical stage (OR = 3.594; 95% CI, 1.828‐7.065; *P* < .001; *I* ^2^ = 71.7%; P_H_ = 0.001), distant metastasis (OR = 4.598;95% CI, 1.411‐14.988; *P* = .011; *I* ^2^ = 78.1%; P_H_ = 0.001), and chemoresistant (OR = 6.400; 95% CI, 2.107‐19.441; *P* = .001; *I* ^2^ = 49.6%; P_H_ = 0.159). However, no correlation between circPVT1 expression and age, gender, histological grade, tumor size, and T stage was detected in our results. Specially, we found two studies of Chen et al and Kong et al on GC might cause oscillation to our results. After omitting the two studies, we detected a significant correlation between increased expression of circPVT1 and tumor size (OR = 2.345;95% CI, 1.466‐3.780; *P* < .001; *I* ^2^ = 0.0%; P_H_ = 0.503) and T stage (OR = 3.614; 95% CI, 1.835‐7.118; *P* < .001; *I* ^2^ = 0.0%; P_H_ = 0.658); and a more significant correlation between high circPVT1 expression and lymph node metastasis (OR = 3.050; 95% CI, 1.909‐4.873; *P* < .001; *I* ^2^ = 30.4%; P_H_ = 0.207) and distant metastasis (OR = 7.560; 95% CI, 3.015‐18.959; *P* < .001; *I* ^2^ = 43.5%; P_H_ = 0.151). Similarly, there was still no correlation between circPVT1 and age, gender, and histological grade.

**TABLE 2:** Meta‐analyses of the correlation between circPVT1 expression and clinicopathological features

Variables	No of studies	No of patients	Model	OR 95%CI *P* value	Heterogeneity *I* ^2^ (%) P_H_
Age (older vs younger)	10	861	Fixed	0.864 (0.650‐1.149) .316	0.0 0.674
Gender (Male vs Female)	9	762	Fixed	1.077 (0.788‐1.471) .641	0.0 0.654
Histological grade (poorly vs well/moderately)	3	366	Radom	0.818 (0.345‐1.942) .649	65.7 0.054
Tumor size (large vs small)	6	574	Radom	1.599 (0.908‐2.815) .104	58.6 0.034
T stage (late vs early)	5	474	Radom	1.263 (0.362‐4.402) .714	85.3 0.000
Lymph node metastasis (positive vs negative)	9	813	Radom	2.019 (1.026–3.976) **.042**	77.5 0.000
Clinical stage (late vs early)	8	718	Radom	3.594 (1.828–7.065) <**.001**	71.7 0.001
Distant metastasis (positive vs negative)	5	483	Radom	4.598 (1.411–14.988) **.011**	78.1 0.001
Chemoresistant (positive vs negative)	2	137	Fixed	6.400 (2.107–19.441) **.001**	49.6 0.159

Abbreviations: OR, odd ratio; 95% CI, 95% confidence interval.

Bold values indicate as statistically significant differences (*P* < .05).

### 3.6 Publication bias and sensitivity analysis

**FIGURE 4:**
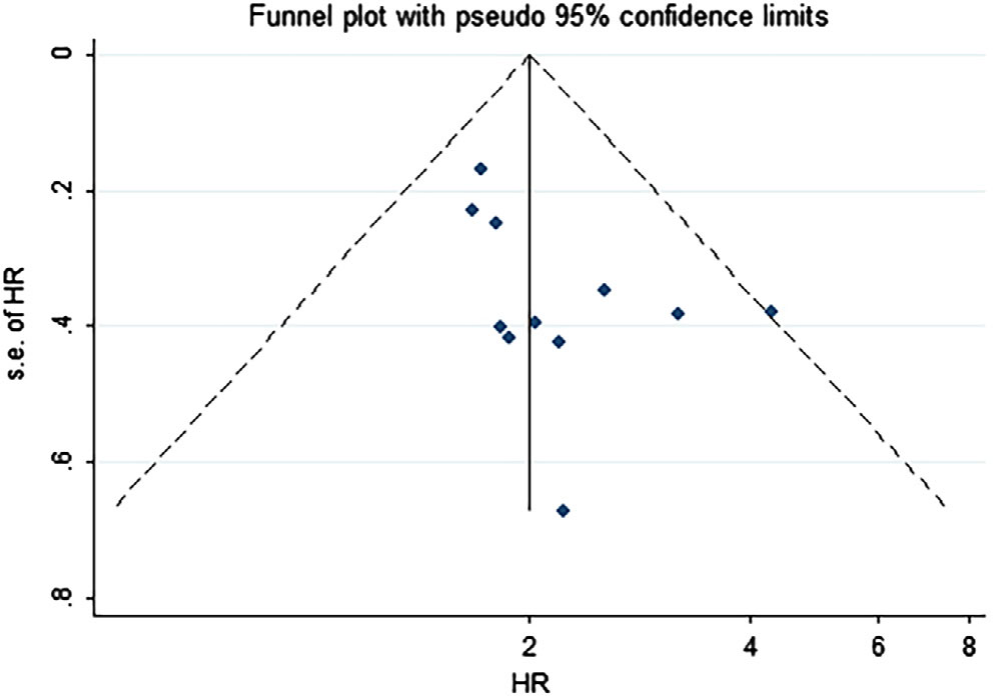
Funnel plot of publication bias based on overall survival

**FIGURE 5:**
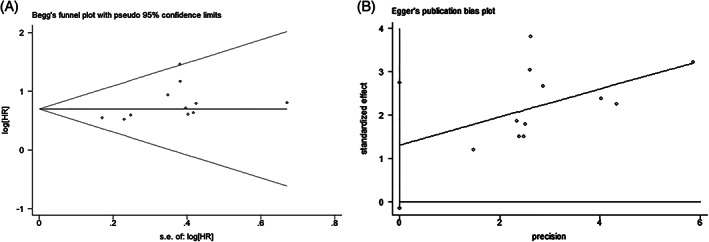
Begg's funnel plot (A) and Egger's publication bias plot (B) of publication bias based on overall survival

**FIGURE 6:**
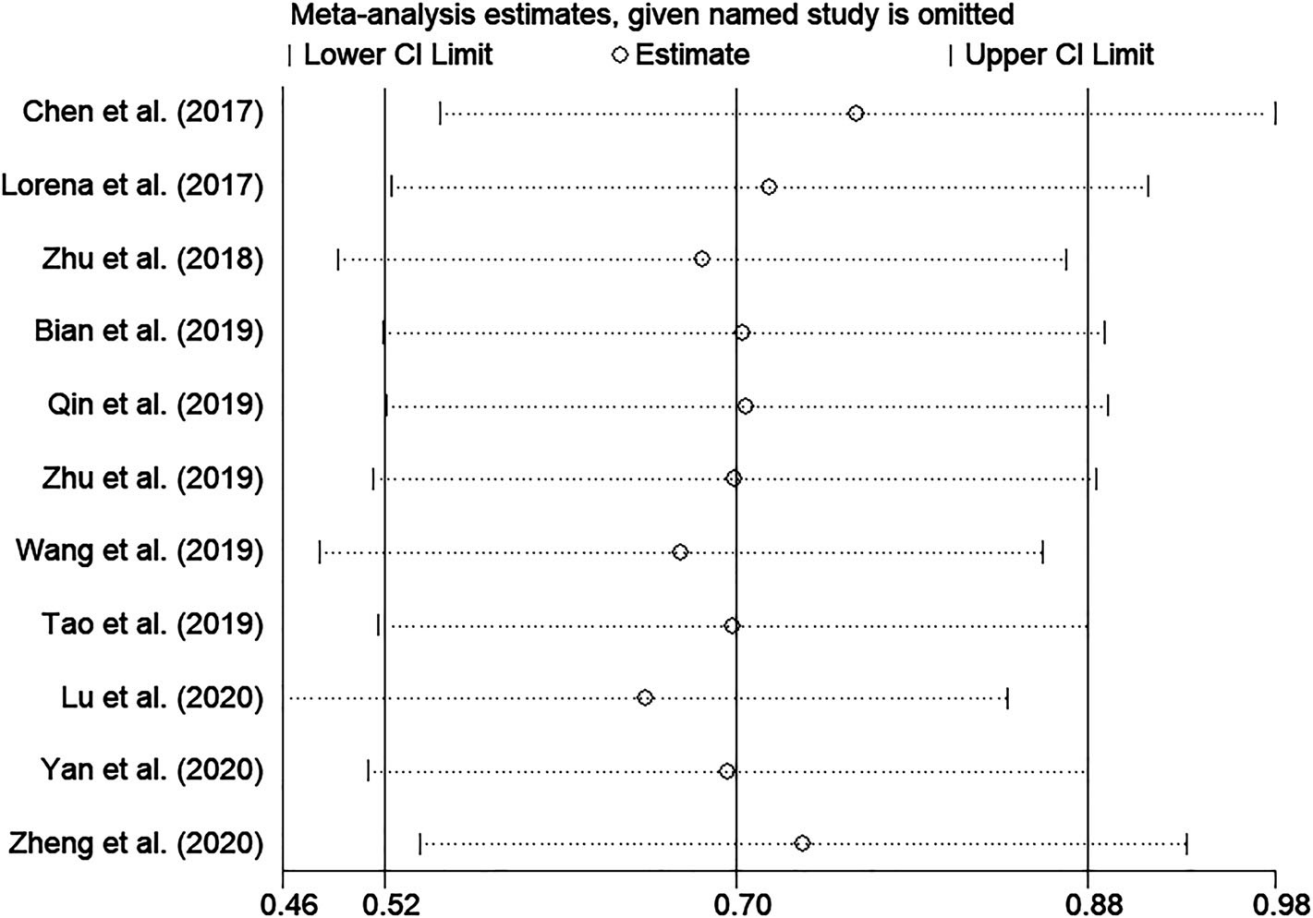
Sensitivity analysis of circPVT1 expression for overall survival

We conducted funnel plot analysis, Egger's, and Begg's tests to assess potential publication bias for OS. The funnel plot of OS revealed no evident asymmetry, indicative of no obvious evidence of publication bias for OS (Figure 4). Meanwhile, Begg's test: *P* = .213 and Egger's test: *P* = .073 also indicated there was no evident publication bias in publications reported OS (Figure 5). The sensitivity analysis was conducted to evaluate the stability of pooled results. After excluding each individual study, there was no significant change on the results, indicating the pooled result was reliable and stable (Figure 6).

## 4 DISCUSSIONS

CircPVT1, as an oncogenic circRNA, has been found to be upregulated in various human cancers, such as GC, CRC, BCa, and osteosarcoma.^15^, ^16^, ^20^ It has been shown that circPVT1 can modulate multi‐aspect processes of tumor development, such as cellular proliferation, metastasis, and chemoresistance. It mainly exerts its oncogenic effects through sponging different miRNAs, thus attenuating their inhibitory effects on downstream targets. For instance, circPVT1 has been found to promote osteosarcoma cells metastasis via directly targeting miR‐526b/FOXC2 and miR‐205‐5p/c‐FLIP, respectively.^23^, ^32^ By downregulating Sirtuin7(SIRT7) via sponging miR‐3666, circPVT1 can promote tumor growth and inhibit cell apoptosis in HCC.^33^ Numerous studies have highlighted that circPVT1 can enhance cancer drug resistance and contribute to worse clinical outcome of cancer patients, indicating that targeting circPVT1 may be a promising therapeutic strategy to overcome chemoresistance and improve prognosis of cancer patients. For example, exosomal circPVT1 has been found to induce cisplatin resistance by stimulating *YAP1* via sponging miR‐30a‐5p in GC in both vivo and vitro.^34^ Via sponging miR‐134p to upregulate the expression of ZEB1, circPVT1 can also promote GC cells resistance to paclitaxel.^35^ In osteosarcoma, circPVT1 has been found to confer multidrug resistance by upregulating ABCB1 expression.^16^ Similarly, circPVT1, which is upregulated in LAD tissues, can promote chemoresistance by inducing ABCC1 via sponging miR‐145‐5p in LAD A549/DR cells in vitro.^21^ Furthermore, emerging evidence suggests that circPVT1 can function as a promising clinical biomarker for human cancer. The diagnostic significance of circRNA in several cancer types, including osteosarcoma, GC, and OSCC, has been validated in some studies.^16^, ^17^, ^36^ Notably, several studies have demonstrated that the expression level of circPVT1 is associated with prognosis and clinicopathological features of various cancers, indicative of the potential role of circPVT1 as an effective predictor for clinical outcomes. However, because some studies have shown contradictory results, the correlation between circPVT1 expression and human cancer prognosis and clinicopathological features is still inexact and inconsistent. Meanwhile, several studies have indicated that circPVT1 expression levels were usually upregulated in advanced stage compared to early stage of cancer progression. This stage‐dependent expression pattern of multiple noncoding RNAs has been detected in various human cancers. For instance, it has been found that hsa‐miR‐181b‐1 and miR‐141 are upregulated in late‐stage of tumor progression of BCa and ovarian cancer, respectively.^37^, ^38^ Therefore, it is challenging to employ these ncRNAs as prognostic biomarkers for cancer patients, and how to effectively screen these ncRNAs including circPVT1 in early‐stage of cancer progression and utilize them as prognostic and clinicopathological biomarkers requires further research and clinical trials. Currently, several studies have assessed the prognostic and clinicopathological value of several dysregulated circRNAs in various cancer types.^39^, ^40^ Another study has also shown that upregulation of both lncRNA PVT1 and circPVT1 is associated with poor survival in patients with different cancers.^41^ However, only a few studies in the literature researching the prognostic value of circPVT1 are included in these studies, and the specific role of circPVT1 as clinical biomarker in human cancers has not been systematically evaluated in these studies. In recent years, multiple studies investigating the prognostic and clinicopathological value of circPVT1 have been published, and we have added these studies in the current meta‐analysis. To our best knowledge, no meta‐analysis has been performed to systematically evaluate the correlation between circPVT1 expression and OS data of patients with human cancers. Therefore, we conducted the first‐time and comprehensive meta‐analysis to evaluate the prognostic and clinicopathological significance of circPVT1 in human cancers and 12 eligible studies were collected in the present study.

In our study, we demonstrated that upregulation of circPVT1 had a significant correlation with poor OS of human cancers. As for the subgroup analysis, the results showed that all subgroup analyses by tumor type, sample size, cutoff value, and data‐obtained method did not have a significant impact on the prognostic value of circPVT1 in human cancer. Regarding the types of tumors, the results suggested that upregulation of circPVT1 was significantly associated with worse OS in bone neoplasms, lung neoplasms, and digestive system neoplasms, respectively. In terms of the association between circPVT1 expression and clinicopathological features, we demonstrated that high circPVT1 expression was remarkably related to lymph node metastasis, late clinical stage, distant metastasis, and chemoresistant. Interestingly, a significant correlation between circPVT1 expression and tumor size, T stage, lymph node metastasis, late clinical stage, distant metastasis, and chemoresistant was detected after omitting the two studies on GC. Therefore, further studies are needed to investigate the relationship between circPVT1 expression and clinical features in GC and other cancer types. In conclusion, the results of the current meta‐analysis indicate that circPVT1 is a promising prognostic and clinical biomarker for various human cancers.

However, several limitations should be taken into consideration in this meta‐analysis. Firstly, most eligible studies in the meta‐analysis were conducted in China and there may exist ethnic bias, thus weakening the representativeness of the study results. Meanwhile, only studies published in English were enrolled in the meta‐analysis, and studies with negative results may not have been published, which may contribute to selection bias and publication bias. Secondly, the number of studies in some major tumor types, such as bone neoplasms, was limited, which may influence the results of subgroup meta‐analysis based on cancer types. Also, there was only one study researching the prognostic value of circRNA in some cancer types, such as HNSCC, PTD, and HCC, so it was difficult to identify the correlation between circPVT1 expression and clinical outcome in those cancers. Thirdly, several studies only provided the survival curve, so the indirectly extracted HRs and 95% CIs may cause some errors and lead to potential source of bias. Fourthly, the cutoff value for distinguishing high or low circPVT1 expression levels was inconsistent, which may account for potential heterogeneity. Finally, the sample size of patients in this meta‐analysis was relatively small, so more studies and large samples would be necessary to better explore the correlation between circPVT1 and prognosis and clinical characteristics in patients of various cancer types.

## 5 CONCLUSIONS

In conclusion, upregulation of circPVT1 is significantly correlated with worse OS in human cancers. This study indicates that circPVT1 can function as a promising prognostic and clinical biomarker, and targeting circPVT1 may be a novel therapeutic strategy for human cancer. Due to the limitations in this meta‐analysis, future experimental research and clinical trials are required to verify the prognostic and clinicopathological value of circPVT1 in human cancers.

## CONFLICT OF INTEREST

The authors declare that they have no competing financial interests.

## AUTHOR CONTRIBUTIONS

All authors had full access to the data in the study and take responsibility for the integrity of the data and the accuracy of the data analysis. *Conceptualization*, Z.L., L.L.; *Data Curation*, Z.L., X.T., L.W., L.L.; *Formal Analysis*, Z.L., X.T., L.W., L.L.; *Investigation*, Z.L., X.T., L.W., L.L.; *Methodology*, Z.L., X.T., L.W., L.L.; *Project Administration*, Z.L., X.T., L.W., L.L.; *Resources*, Z.L., X.T., L.W., L.L.; *Software*, Z.L., X.T., L.W., L.L.; *Validation*, Z.L., L.L.; *Visualization*, Z.L., X.T., L.W., L.L.; *Writing‐Original Draft*, Z.L., X.T., L.L.; *Supervision*, L.L.; *Writing‐Review & Editing*, L.L.

## ETHICS APPROVAL AND CONSENT TO PARTICIPATE

Not applicable.

## Data Availability

The data sets used and/or analyzed during the current study are available from the corresponding author on reasonable request. The data that support the findings of this study are openly available in PubMed, Embase, Web of Science and Cochrane Library online databases, Reference numbers 1‐41.
